# Children and Homœpathy

**Published:** 1914-03-28

**Authors:** J. Roberson Day

**Affiliations:** Hon. Physician to the Children's Homœpathic Dispensary


### The Children and Homœopathy.

*To the Editor of* The Hospital.

Sir,—I am interested to see in your issue of March 21, 1914, the paragraph, "Lord Wemyss on Homœopathy." Your readers may like to know of the existence of the Children's Homœopathic Dispensary at Shepherd's Bush, recently opened. An inaugural meeting was held, by the kind invitation of Lady Perks, last June, when the needs of a Children's Homœopathic Dispensary for London were recognised. The many advantages which homœopathy offers to children are well recognised, and this new institution promises to supply a long-felt want. It is conducted as an out-patient department of a general hospital. The medical staff are honorary, and there are special departments for medical, surgical, eye, ear, nose, throat, and skin cases. Many of the staff and committee are connected with the London Homœopathic Hospital. Funds are urgently needed for the development of this growing institution, and will be thankfully received by the Hon. Secretary, Shepherd's Bush Green. Sir George Wyatt Truscott, Bart., who is hon. treasurer, has shown the greatest interest in the work since its inception.—Yours faithfully, J. Roberson Day, M.D. (Lond.),
Hon. Physician to the Children's
Homœopathic Dispensary.

